# Organisational barriers to the facilitation of overseas volunteering and training placements in the NHS

**DOI:** 10.1186/s12913-018-2853-z

**Published:** 2018-01-31

**Authors:** John Chatwin, Louise Ackers

**Affiliations:** 1 School of Health and Society, University of Salford, Room C530 Allerton Building, Salford, M6 6PU UK; 2 School of Health and Society, University of Salford, Room C501 Allerton Building, Salford, M6 6PU UK

## Abstract

**Background:**

Undertaking a period of voluntary work or a professional placement overseas has long been a feature of medical training in the UK. There are now a number of high profile National Health Service (NHS) initiatives aimed at increasing access to such opportunities for staff at all levels. We present findings from a qualitative study involving a range of NHS staff and other stakeholders which explored barriers to participation in these activities.

**Methods:**

A grounded theory methodology was drawn upon to conduct thematic based analysis. Our data included in-depth, semi-structured interviews with a range of returned volunteers, non-volunteers and other stakeholders (*n* = 51) who were, or had been, employed by the NHS.

**Results:**

There are significant barriers to placement and volunteering activity stemming from structural and organisational shortcomings within the NHS. Difficulties in filling clinical roles has a significant impact on the ability of staff to plan and undertake independent placements. There is currently no clearly defined pathway within the NHS by which the majority of grades can apply for, or organise, a period of overseas voluntary or professional placement activity. There were divergent views on the relevance and usefulness of overseas professional placements.

**Conclusions:**

We argue that in the context of current UK policy initiatives aimed at facilitating overseas volunteer and professional placement activity, urgent attention needs to be given to the structural and organisational framework within which such initiatives will be required to work.

**Electronic supplementary material:**

The online version of this article (10.1186/s12913-018-2853-z) contains supplementary material, which is available to authorized users.

## Background

Undertaking a period of voluntary work or a placement overseas has long been a feature of medical training in the UK, and the option to participate in such activity is built in to a wide variety of clinical training programmes [1]. Until recently, providing such opportunities for the myriad of other grades and roles within the National Health Service (NHS), particularly non-clinical ones, has not been a priority, and employment structures within the organisation have remained relatively inflexible in this respect [2]. The potential value that even short periods within foreign healthcare and cultural contexts can bring to both individual employees, and the wider NHS is now being more broadly promoted, [3] and there are currently a number of high profile healthcare training initiatives in the UK aimed at increasing the availability of, and access to, overseas volunteering and placement opportunities for staff at all levels [4–6].

The value of training placements and volunteering activities abroad are widely debated and difficult to quantify. This is a major issue for the NHS in the context of an ever worsening financial and human resource crisis. The vast range of potential locations, organisational focus and methods of assessing impact mean that it can be difficult to draw meaningful conclusions about the effects that activities have, both on volunteers, and the communities in which they are embedded. There are likely to be significant differences, for example, between the experiences and learning outcomes reported by short-term volunteers versus longer-term volunteers. The settings in which people are deployed (e.g. developmental settings in stable contexts; humanitarian aid in unstable locations; emergency responses to natural disasters and so on), will also have a bearing on the experience and perceptions of volunteering.

The potential benefits to the NHS claimed for volunteering and placement activities abroad include the entrenchment of the organisation’s core values - the so-called ‘6 Cs’: care, compassion, competence, communication, courage and commitment [7]. Mutual learning and knowledge mobilisation benefits are now also being recognised [8–12], but there may be other, more tangential payoffs that result from participation [13]. These include the grab bag of assorted ‘soft skills’ such as social and cultural awareness, leadership and team working which are so often reported by returning volunteers, even though as with the ‘6 Cs’, there is little consensus on exactly what these terms actually mean [14–16]. Many researchers, for example, have described the personal and psychosocial impact of overseas experience (see, for example, [17–19]). However, Sherraden et al., [20] highlights the way in which the overwhelming majority of studies in this area cite positive effects on volunteers but rarely access the potentially negative effects. Consequently, the mechanisms by which experiences actually lead to ‘gain’ or ‘loss’, at whatever level, are poorly understood. The analysis we present here focuses on aspects of the qualitative component of a mixed methods study *Measuring the Outcomes of Volunteering for Education* (MOVE) (see Acknowledgements), which aimed to explore the issues and context around international placements skills gain, and describe core personal and professional skills outcomes that are directly relevant to the NHS.

MOVE consisted of two distinct, yet interwoven thematic strands that incorporated both qualitative and quantitative approaches [21]. The quantitative strand of the project aimed to develop a psychometric tool that could be used by placement providers to match staff with specific types of overseas activity. While the parallel qualitative theme was concerned with exploring issues around 1) the impact that professional volunteering had on volunteers themselves. 2) the specific types knowledge and skills that returning volunteers gained, or perceived themselves to have gained. 3) barriers to participation.

In this article we are concerned particularly with the barriers theme. In the context of an inclusion programme that is already underway, we focus on elements that might hold NHS staff back, or discourage them from taking up a placement opportunity abroad. For example, are particular staff grades more likely to engage in this type of activity than others? What impact do working practices and established employment systems within the NHS have on people’s experiences – both of accessing placement opportunities, and of integrating these with their work or training?

## Methods

This was a qualitative interview based study which utilised the principles of grounded theory [22]. We worked with thematic and narrative analysis to develop a plausible representation of the world view in which our participants were embedded, and engaged in a research strategy which incorporated ongoing analysis, comparison and theorizing. Our interview schedules (i.e. questions we asked and the themes we chose to explore) were individually tailored to each interviewee and continually reviewed and revised as necessary on the basis of emerging evidence (see Additional file 1 for basic interview guide). In the context of this study, this meant talking to staff and stakeholders who were not actively engaged in professional volunteering, as well as those who were. As a starting point, we asked participants who had been volunteers to talk about their personal experiences while away, and the process they had followed in organising their trip; we asked them about the impact they felt they had made on the communities that had hosted their visit, and of the impact the experience had had on them personally; we explicitly asked about any barriers they had encountered throughout the process – both at an organisational level, and also while they were away. For non-volunteers, the emphasis was more on their perceptions of the value of volunteering in the context of the NHS and their role within it; whether or not organisational barriers had been a factor in discouraging them to volunteer, for example.

## Results

Our primary corpus was comprised of in-depth, semi-structured interviews with a range of returned volunteers, non-volunteers and other stakeholders (*n* = 51) who were, or had been, employed by the NHS. These data were collected during 2014 and 2015 and participants were purposively sampled to broadly reflect the current proportions of staff cadres current represented within the organisation. The sample consisted of: qualified or trainee doctors (*n* = 11); nurses and midwives (*n* = 16); clinical support staff (*n* = 6); managerial and administrative staff (*n* = 10); and ‘others’, including ambulance and maintenance staff etc. (*n* = 8). 8 respondents (15%) had not been on a placement or had no overseas experience. We initially recruited participants via NHS contacts based at the University of Salford (School of Nursing, Midwifery, Social Work and Social Science), and the University of Manchester Medical School. A significant proportion of volunteer and ex-volunteer respondents were contacted via established provider organisations that currently offer overseas placements to NHS staff. We then utilised a selective snowballing technique to widen our recruitment pool and reduce bias towards any particular group or sector.

Although in this article we are concerned primarily with interview data, the wider MOVE study incorporated a range of other relevant material. This included personal diaries; blogs and similar written accounts; assessment and evaluation data from placement organisations; pictures, video and other media; local and national policy documentation.

Ethical approval was obtained from the University of Manchester Research Ethics Committee, and the University of Salford Research Ethics Committee. Written consent was obtained from participants.

## Discussion

It needs to be acknowledged that the current drive in UK policy towards providing greater opportunities for professional volunteering within the NHS are not the result of any kind of systematic organisational analysis [12], and are still a relatively contentious issue. However, leaving aside broader arguments over whether or not they are a worthwhile endeavour, we work from a position in which such initiatives are already underway. It is well established that the staff cadres currently undertaking the majority of overseas professional placement and voluntary activity are ‘medical and dental’, and ‘nursing, midwifery and health visitor’ [21]. Dowel and Merrylees [23], report that an estimated 40% of medical students seek out experiences working in resource-poor settings during their training and a recent large scale NHS staff survey by Chatwin and Ackers [22] indicates that around 21% of nursing and midwifery staff have volunteered or conducted a placement overseas. Understandably, considering that the NHS is the largest and most complex organisation in Britain [24] it emerged that significant barriers to placement and volunteering activity stem from structural and organisational shortcomings. Staffing issues within the NHS – particularly focusing on shortages among nursing and clinical grades - are once again an active political issue [25]. However, the problem is nothing new [26, 27], and it appears that difficulties in filling clinical roles has had a consistent impact on the ability of staff to plan and undertake independent placements. We found that, particularly in the case of nursing and midwifery staff, the underlying perception was that attempting to take periods of extended leave would create significant difficulties, and ultimately prove to be not worth the trouble. Exceptions to this do occasionally arise – such as nationally co-ordinated calls for volunteers to assist in urgent health crises abroad. However, the organisational structures put in place to respond to such events are by their nature temporary [28].

For many staff, access to employment relevant training and placement opportunities - let alone those which might involve extended periods abroad - is already difficult. While for the majority of professional grades within the NHS, continuing professional development (CPD) can be a cornerstone of the support offered by the organisation, much CPD is highly specialised and integrated into the ongoing demands of day to day activity, and this is true across all staff cadres. A key feature of this type of training is that it is very easily codified. It is straightforward to accurately test and measure the extent to which a person has learned from their training. But in the case of an overseas placement, things can be different. Placements and training that demand more than a short period away from routine employment, or that cannot be integrated directly into on-the-job training can bring with them a whole raft of access issues. These range from simply being able to square a period away with home and work commitments, through to trickier issues of justifying the cost and benefits that an employer (i.e. the NHS) can expect as a result of their trip. This is not so much of a concern if a person simply has altruistic motivations and is organising a trip independently in their own time. But if there is a need for some or all of the cost of their outing to be met as part of their CPD, things become more complicated.

For qualified staff (i.e. not those who are in training) there is currently no clearly defined pathway within the NHS by which the majority of grades can apply for, or organise, a period of overseas voluntary or professional placement activity. Practical advice on how to engage with the relevant administrative processes is difficult to come by [29] and for many, the utilisation of informal networks and personal contacts proved to be the most effective route:

*‘It’s almost like an underground type of thing. It’s going on everywhere but not a lot of people know about it. And I have been to a couple of conferences and you know it’s so surprising how many people are involved in it.’ (Nurse manager)*

For many staff, the easiest way of breaking through bureaucratic barriers may be to just use annual leave entitlement. Although this approach is only really suitable for relatively short periods abroad, and can be risky in terms of official support structures, insurance and so on, we found it was very common:

*‘I didn’t get any allocated time so I arranged to use my holiday time, and I did get a lot of resistance from my line manager. She did not agree with what I was doing, not at all and I just thought I wanted to go. I had to say I am willing to use my own time, I don’t want anything else, and she was being very awkward so I just started using my own time. That was the resistance I got, and I do think there is a lot of that.’ (Midwife trainer)*

One departmental manager, who regularly organised short trips for her UK colleagues to undertake emergency obstetric training in Africa, also highlighted that even when there is relatively good access to placement opportunities within a given department, there can still be micro-political issues to overcome:

*‘ . . .being released from work, it’s a bit difficult politically. I mean even in our small group. I manage a group of educators - probably eleven or twelve and of those it can cause political…..you know just to offer a place, and who is going to go next, when you can go, who allowed them to go?’ (Medical trainer)*

From a line management perspective, the real issue was often not whether or not people would benefit from a placement opportunity - the majority of managers we spoke to acknowledge the potential benefits to staff of overseas experience, even if they had not been themselves. The issue was primarily one of providing cover, and where funds for this cover would come from:

*‘I can’t afford to back them to go, so even if they financed themselves they need to back fill their position. Like if you come to me and say you would like to go to Uganda and take two weeks annual leave, but I still, as head of midwifery, have to call off your shifts for weeks so I would say the financial situation will be absolutely no. So [you] come back to me and say I have got some funding from [volunteering charity] and they are gonna pay for four weeks, then as long as they gave me the money then I would let you go. It’s two weeks, maximum three weeks at an absolute push for annual leave.’ (Department manager)*

Even where processes were in place to offer direct payment to departments while staff were away – as is the case when staff are seconded for duty with organisations such as the Army Reserves, the disruption this causes can be difficult to manage. It would appear then that currently, it is at mid-level line management where significant (if largely unintended) barriers are encountered. Respondents who were managers themselves, or who were familiar with staffing and recruitment processes within the NHS, pointed out that if large scale initiatives are rolled out, the practical problem of designing programmes that are effectively integrated and ‘process friendly’ will need serious attention.

*‘[In providing placements] it is about remembering two groups. It is all about the senior managers - and that is like pushing against an opened door – they see exactly what it’s about. . . but on a practical level it’s the middle manager, the line manager. That is a much tougher nut to crack. . it is about information, and it is about getting information out there.’ (Senior manager, external placement provider)*

The co-ordination of what could potentially be very large and complex national placement programmes within existing NHS organisational structures faces another problem; that of regional inconsistency. At a national level, the NHS has a well-established and coherent organisational framework [30]. However, our respondents were affiliated to a variety of different NHS trusts and administrative regions, and it was clear that structural and organisational conventions varied widely depending on the idiosyncrasies of local policy. There was similarly a degree of variation between hospitals and community based organisations within regions, and even between departments within hospitals. At one level these inconsistencies have been compounded by periodical adjustments to the management structure. In particular, efforts such as those made during the late 1990’s to devolve decision making processes to a more local level [31]. The ‘flatter organisation’ that has resulted may now be having the incidental effect of making national directives, such as the ones we are concerned with, difficult to implement.

We found that although there has been a general improvement of access to placement opportunities across the NHS over recent years, the situation is still relatively piece meal. If the proposed expansion in volunteer and placement opportunities is to develop, we would suggest that there is a clear need for a centralised system to co-ordinate both the allocation of approved placements and the standardisation of cover provision procedures. Althought there have been attempts to provide such services already; notable examples being the International Health Links Centre, based at the Liverpool School of Tropical Medicine, [32] and the ‘Global Health Exchange’ (GHE) based in Salford [33], there is a gap between the centralised facilitation they are aiming to provide for clinical staff, and that which might become available for lower or non-clinical grades. Similarly, as we have emphasised already, such affiliated organisations are still essentially external to NHS employment structures and do not have the level of integration which would allow them to make a useful impact on the organisation as a whole.

Overseas experience is seen as an opportunity to capitalise on a range of potential learning resources, and not only those that build on clinical skills. However, despite the current rhetoric of inclusion (see, for example, [33]) the Department of Health Framework for International Development [12] only contains case study examples from doctors, nurses and physiotherapists. These groups represent the three main NHS staff cadres that already engage in the majority of placement activities [21]. Significantly, at this stage there is still no mention of the myriad of other roles which make up the rest of the NHS workforce - the administrators, porters, IT operatives, catering staff and so forth.

With the underlying assumption that placement opportunities could potentially become available to all staff, we asked respondents about how they thought such a system could operate, and what might discourage them from engaging with it. Offering everyone an overseas placement was regarded by many as basically unworkable. This attitude was particularly evident from non-clinical staff, and there were even concerns over formal and informal coercion (i.e. at senior and line management level) if placement schemes became target driven.

*‘I’d never really considered [volunteering abroad] as part of my career path. It’s surprising the NHS are rolling this out to all job types. There’ll be a lot of potential problems with people fitting it in. Will they feel pressured to go on a placement? Lots of people have no interest or inclination to do this type of thing.’ (Non-clinical administrator)*

We specifically asked respondents whether or not they thought that volunteering and overseas placements were something that all NHS employees would find useful. Here, there was a demarcation between clinical and non-clinical grades – essentially formed along socio-demographic lines. While at an abstract level, placement and voluntary activity was regarded as useful and worthwhile, for some respondents it was not conceived of as something particularly relevant to their own working life. The perception was often that activities abroad were primarily for the benefit of the host country (i.e. altruistic) rather than something that might actively add to a person’s continuing professional development.

*‘[The situation in poorer countries] is completely different. I think it is more beneficial for people from poor countries to come here, but I’m not sure how beneficial it is for us to go there really. It’s beneficial for them.’ [Hospital maintenance worker]*

This was increasingly evident the further removed from the clinical grades a person’s role was. Responses from staff who had not had overseas experience were sometimes ambiguous, and appeared to reflect an underlying view of volunteering as a kind of privileged activity, available only to those who had a role that allowed for a degree of freedom:

*‘It doesn’t seem to be offered to people [theatre assistants] in the operating theatres cos we’re on the coal face doing the important work [laughs]. . . Value? Possibly, possibly not. I’d love to go abroad and see how other people work, but value – possibly not. I’ve spoken to people who’ve gone abroad and they don’t seem to bring very much back with them to be quite honest. They tell you how – people who have been to Africa or India – they come back and they say it’s been great for them to see how other people work. But the only thing they seem to bring back is that they’re really happy to be back and they’re not working in those conditions anymore.’ (Operating theatre technician)*

Barriers to inclusion may be deeply engrained and psychosocial in nature, but at a practical level too, the majority of employees do not have the level of flexibility that doctors, and to a lesser extent, nurses and other clinical professionals enjoy. Psychological barriers exist that have developed from assumptions about why particular staff groups might want to volunteer or undertake an overseas placement. Again, these tend to broadly reflect a person’s position in the NHS employment hierarchy. Many of the trainee doctors we spoke, for example, were open about the – sometimes quite instrumental – reasons why they chose to conduct a placement overseas. These might relate to learning specific clinical skills that they would not necessarily have chance to practice in the UK, but they could also be connected to CV building and career development. ‘Lower’ grades, however, could be less strategic in their approach and it should be acknowledged that as a wider range of staff are asked to think about the prospect of a placement opportunity, there will be large sectors of the NHS workforce who do not immediately see this as a useful addition to their ongoing professional development. As initiatives progress, attention will need to be paid not only to the basic provision of placement opportunities, but also to raising general awareness about the value that they might represent to groups who would not routinely engage with them. This in itself may prove to be one of the key barriers to greater integration.

## Conclusions

Our study was conducted in the context of initiatives within the NHS which are already gathering some momentum. A key finding was the urgent need for the development of a system of standardised placement opportunities integrated across the entire organisation. Whilst there is evidence to support the view that volunteering and international placements present valuable and enjoyable opportunities for health professionals at all levels, it may be that in reality this is currently insufficient to justify more widespread NHS expenditure in this area. In the spirit of widening participation and the removal of organisational barriers, the quality of learning and potential for innovation needs to be more directly quantified, and aligned with specific role needs. Every international volunteering or placement experience is distinct in terms of its context, the activities the professional volunteer engages in and the learning opportunities these present.

The piecemeal arrangements which are currently in place are unlikely to function efficiently (or at all) if challenged by a significant rise in demand. We acknowledge that moves to centralise processes are underway. However, we found that mid-level line-management is currently a major bottle neck in terms of access to placement opportunities. The situation is exacerbated by the lack of regional standardisation across different NHS trusts and even within departments. More attention needs to be given not only to the provision of new placement opportunities, but also to raising awareness about the value that these opportunities might have to staff groups who do not currently regard them as relevant. In order to reach these groups it may be useful to focus on ensuring that broader aspects of ‘value’ are more clearly highlighted.

## Additional file

Additional file 1: Interview schedule / themes. (PDF 672 kb)

## Abbreviations

CPD: Continuing professional development
GHE: Global Health Exchange
HEE: Health Education England
MOVE: Measuring the Outcomes of Volunteering for Education
NHS: National Health Service
