# Supplementary material for: Organisational barriers to the facilitation of overseas volunteering and training placements in the NHS
Source: BMC Health Serv Res. 2018 Jan 31;18:69. doi: 10.1186/s12913-018-2853-z (PMC5793435; doi:10.1186/s12913-018-2853-z)
Supplement: Additional file 1: — Interview schedule / themes. (PDF 672 kb) [file 12913_2018_2853_MOESM1_ESM.pdf]

<table>
<tr><td>Participant No.</td><td colspan="2">M___ /_____</td><td>Volunteer: Y ☐ N☐</td></tr>
<tr><td>Participant Name:</td><td colspan="2"></td><td>Gender: M ☐ F☐</td></tr>
<tr><td>Date of Birth.</td><td></td><td colspan="2">single ☐ married ☐ living with partner ☐</td></tr>
<tr><td>Role / Grade</td><td colspan="3"></td></tr>
<tr><td>Ethnicity.
(see code sheet)</td><td colspan="3"></td></tr>
<tr><td>Dates of volunteering
(if applicable)</td><td colspan="3"></td></tr>
</table>

Introduction:

Go over the purpose of the study with participant.
Check they are still willing to take part.
Check they are happy for interview to be recorded.
Prompt for and answer any other queries.
If relevant, ask them to fill in the consent form.

Interview themes

	Introduction
**1)**	**General intro questions (not required if SVP and volunteer / background available already).**<br><br>Where did you go on your international placement?<br><br>How did you decide to go there?<br><br>What happened to you to prepare you to go there?<br><br>What happened when you arrived?<br><br>Where you with others or on your own?

	**Section 1 – Impacts while on placement**
**2)**	**Impact of being in Uganda / overseas volunteer placement:**<br><br>**What impact did your placement / time abroad have on you?**<br><br>**Prompt for**: experiences, expectations, gains, losses, personal growth, professional growth.
**3)**	**What impact do you think your placement / time abroad had on your patients?**<br><br>**Prompt for**: sustainability, training, specific examples of care related events.
**4)**	**What impact do you think your placement / time abroad had on your colleagues?**<br><br>Prompt for: co-presence and team working.
**5)**	**Do you think your time abroad had any impact on local systems?**<br><br>**Prompt for:** micro / macro effects – i.e. interpersonal / wider organisational<br><br>**Prompt for**: Engagement with other NGOs In country, mobility & knowledge<br><br>**Prompt for**: Volunteer congestion & swamping
	**Section 2 – Impacts now you're back in the UK**
**6)**	**Now that you're back in the UK, how is your experience influencing you?**<br><br>**Prompt for:** possible career effects, personal changes, attitudes towards care, attitudes towards other staff etc.
**7)**	**How has your experience influenced the way you deal with patients?**<br><br>**Prompt for:** specific examples of change (positives + negatives)

8)	**Following your experience abroad as a volunteer, how have you found working with colleagues back in the UK?**<br><br>**Prompt for:** specific examples (positives + negatives), difficulties.<br>**Prompt for:** specific examples of system related issues (micro / macro)
9)	**How would you do things differently if you went again?**<br><br>**Prompt for**: Volunteer role, organisational issues, mentoring issues, cultural issues.
10)	**Overall, would you say your placement (or overseas placements in general) are something the NHS should be encouraging?**<br><br>**Prompt for:** specific reasons why / why not.
